# Novel ROCK Inhibitors, Sovesudil and PHP-0961, Enhance Proliferation, Adhesion and Migration of Corneal Endothelial Cells

**DOI:** 10.3390/ijms232314690

**Published:** 2022-11-24

**Authors:** Kyung Wook Kim, Young Joo Shin, Sammy Chi Sam Lee

**Affiliations:** 1 Department of Ophthalmology, Hallym University Medical Center, College of Medicine, Hallym University, Seoul 07441, Republic of Korea; 2 Hallym BioEyeTech Research Center, Hallym University College of Medicine, Seoul 07441, Republic of Korea; 3 pH Pharma Co., Ltd., B-1009, U-Space, 670 Daewangpangyo-ro, Bundang-gu, Seongnam-si 13494, Republic of Korea

**Keywords:** sovesudil, novel ROCK inhibitor, regeneration, corneal endothelial cells

## Abstract

The loss or dysfunction of human corneal endothelial cells (hCEnCs) is a leading cause of blindness due to corneal failure. Corneal transplantation with a healthy donor cornea has been the only available treatment for corneal endothelial disease. However, the need for way to regenerate the CEnCs has been increased due to the global shortage of donor corneas. The aim of the study is to investigate whether novel Rho-kinase (ROCK) inhibitors can induce the cultivation and regeneration of hCEnCs. Cultured hCEnCs were treated with Y-27632, sovesudil, or PHP-0961 for 24 h. Cellular responses, including cell viability, cytotoxicity, proliferation, and Ki67 expression with ROCK inhibitors were evaluated. We also evaluated wound healing and cell adhesion assays. Porcine corneas were used ex vivo to evaluate the effects of Y-27632, sovesudil, and PHP-0961 on wound healing and regeneration. We performed live/dead cell assays and immunofluorescence staining for SRY (sex determining region Y)-box 2 (SOX2), β-catenin, and ZO-1 on porcine corneas after ROCK inhibitor treatments. Cell viability, cell proliferation rate, and the number of Ki67-positive cells were higher in Y-27632, sovesudil and PHP-0961 treated cells compared to the control. There was no difference in LDH cytotoxicity test between any groups. Cells treated with Y-27632, sovesudil and PHP-0961 showed faster migration, wound healing, and cell adhesion. In the porcine ex vivo experiments, wound healing, the number of live cells, and SOX2-positive cells were higher in Y-27632, sovesudil and PHP-0961 treated corneas. In all experiments, sovesudil and PHP-0961, the novel ROCK inhibitors, were equal or superior to the results of the ROCK inhibitor positive control, Y-27632. In conclusion, sovesudil and PHP-0961, novel ROCK inhibitors have the capacity to regenerate hCEnCs by enhancing cell proliferation and adhesion between cells.

## 1. Introduction

Corneal endothelial cells (CEnCs) are located on the inner surface of the cornea and play an essential role in keeping corneal transparency by pumping out water from the corneal stroma [1]. Reduction in the number of human CEnCs (hCEnCs) due to injuries causes permanent corneal edema [2]. Until now, hCEnCs have been considered unable to proliferate in vivo [2]. Corneal transplantation has been considered the only treatment option for hCEnC diseases [3]. However, there is a global shortage of corneal donors and the survival time from cornea acquisition to transplantation is relatively limited [4]. Thus, it is necessary to develop a new treatment or a new strategy to alleviate the lack of required donor corneas. Furthermore, stem cells for hCEnCs have been found in the transition zone between the trabecular meshwork and the cornea and the possibility of inducing regeneration of hCEnCs in Fuchs corneal endothelial dystrophy (FECD) has been suggested [5], although severe damage to hCEnCs results in bullous keratopathy requiring corneal transplantation [6]. The development of a new treatment strategy has been suggested to protect and regenerate hCEnCs in vivo. 

Rho-associated protein kinase (ROCK) inhibitors were developed as drugs to lower intraocular pressure [7], but it has been suggested to have an effect in hCEnCs [8,9]. It has been reported to promote the adhesion of cultured hCEnCs to the stroma in bullous keratopathy [10] and to promote wound healing after transcorneal freezing in FECD [11]. Ripasudil, another ROCK inhibitor, has a corneal endothelial protective effect in cataract surgery for patients with low hCEnC density [9]. However, both preclinical and clinical studies that have evaluated ROCK inhibitors have reported conjunctival hyperaemia at high incidence rates, therefore the therapeutic potential of most ROCK inhibitors is limited by adverse effects.

Sovesudil, a novel ROCK inhibitor, has been developed to improve ROCK affinity and to degrade rapidly to reduce side effects such as conjunctival hyperemia [7]. Sovesudil, like other soft drugs and are designed to undergo metabolic inactivation by controlled conversion of the active parent molecule into a predictable, nontoxic metabolite [12,13]. ROCK is an effector molecule of RhoA signaling and have the multi-functions including the effect on cytoskeleton and stemness [14,15]. ROCK inhibitors are thought to contribute to the regeneration of hCEnCs by reducing oxidative stress and senescence, inducing mesenchymal-epithelial transition (MET), and inhibiting transforming growth factor-beta (TGF-β) signaling [16,17,18]. In this study, we investigated whether a novel ROCK inhibitor, sovesudil, has the capacity to regenerate hCEnCs. 

## 2. Results

### 2.1. Effect on Cell Proliferation

**Figure 1:**
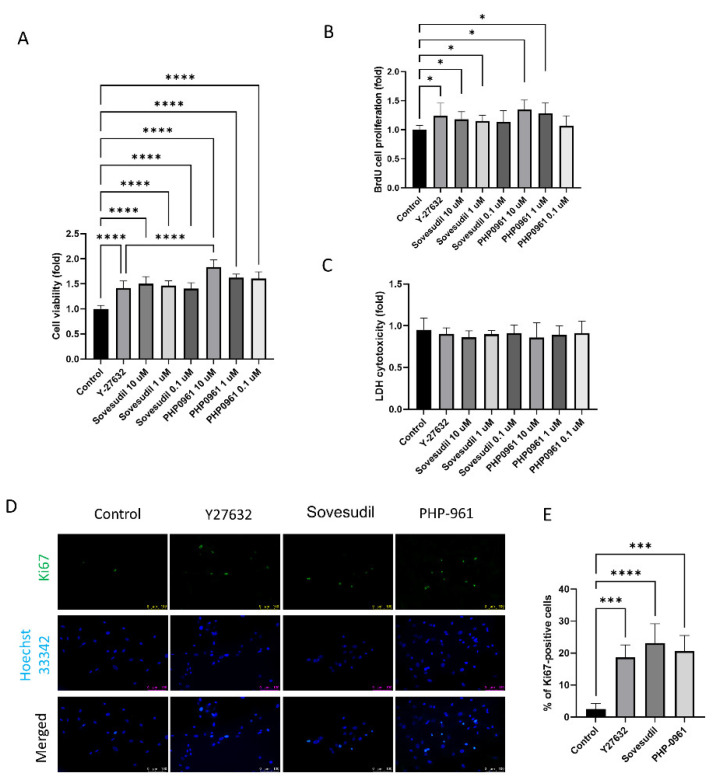
Cell proliferation and cell cytotoxicity. (**A**,**B**) Cell viability and BrdU cell proliferation rate were evaluated by CCK-8. (**C**) Cytotoxicity was measured by the LDH cytotoxicity test. (**D**,**E**) Cell proliferation evaluated by immunofluorescence staining of Ki67, a proliferation marker. * *p* < 0.05, *** *p* < 0.001 and **** *p* < 0.0001.

Cells were treated with Y-27632, sovesudil, and PHP-0961. To evaluate the effect of Y-27632, sovesudil, and PHP-0961on cell proliferation, cell viability and BrdU incorporation assay were performed. Cell viability and BrdU incorporation rate was higher in Y-27632, sovesudil and PHP-0961 and it was highest in PHP-0961 (Figure 1A,B). To assess the cytotoxicity of Y-27632, sovesudil, and PHP-0961, LDH cytotoxicity tests were performed. There was no difference in LDH cytotoxicity test between any groups (Figure 1C). Immunofluorescence staining of Ki67, a proliferation marker, was shown in Figure 1D. Ki67-positive cells increased in Y-27632, sovesudil and PHP-0961 compared to the control (Figure 1E).

### 2.2. Cell Migration and Wound Healing

**Figure 2:**
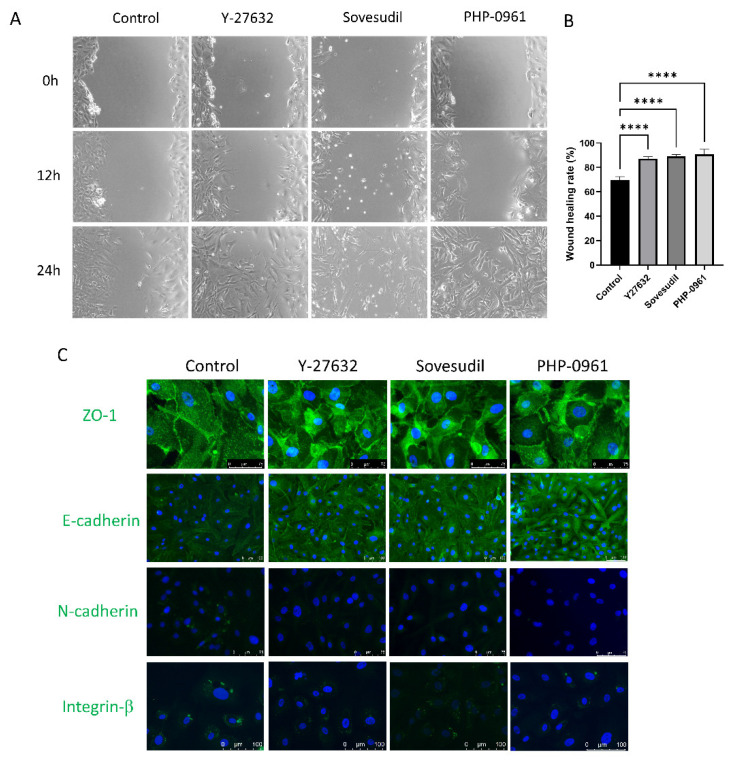
Cell migration and cell adhesion molecules. (**A**,**B**) A cell scratch assay was performed to evaluate the effect of ROCK inhibitors on wound healing rate. (**C**) Cell adhesion molecules were evaluated by immunofluorescence staining for ZO-1, E-cadherin, N-cadherin and integrin β1. **** *p* < 0.0001.

To evaluate the effect of Y-27632, sovesudil and PHP-0961 on wound healing, scratch assay was performed. The wound healing of cultured hCEnC at 12 h and 24 h after scratch is shown in Figure 2A, and the cells treated with Y-27632, sovesudil, and PHP-0961 showed faster migration and wound healing (Figure 2B). Immunofluorescence staining of ZO-1, E-cadherin, N-cadherin and integrin-β1 were performed to investigate the distribution of cell adhesion molecules (Figure 2C). Intracellular localization of ZO-1 was shown in Y-27632, sovesudil and PHP-0961-treated cells. The expression of E-cadherin was increased and N-cadherin and integrin-β1 was not detected in Y-27632, sovesudil, and PHP-0961.

### 2.3. Effect on the Cell Adhesion Assay

**Figure 3:**
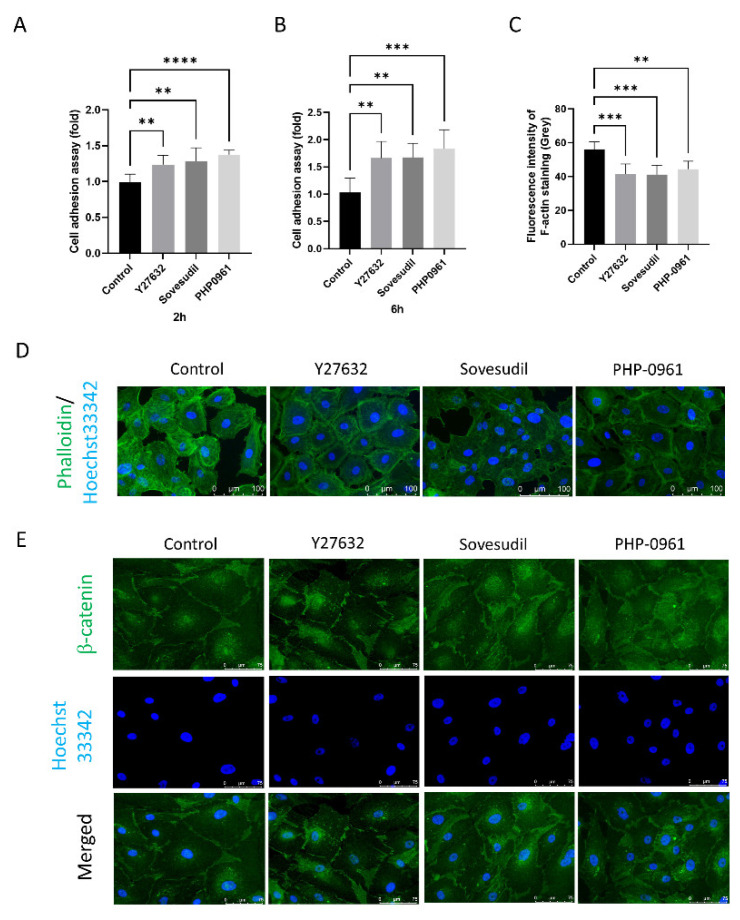
Cell adhesion assay. (**A**,**B**) Cell adhesion was evaluated by crystal violet assay at 2 h and 6 h. (**C**,**D**) Phalloidin staining was performed to evaluated F-actin pattern and fluorescence intensity of F-actin. (**E**) β-catenin was evaluated by immunofluorescence staining. ** *p* < 0.01, *** *p* < 0.001 and **** *p* < 0.0001.

Cell adhesion was evaluated using Crystal violet assay. Cell adhesion was higher in Y-27632, sovesudil and PHP-0961 both at 2 h and 6 h compared to control (Figure 3A,B). Cytoskeleton pattern was evaluated using phalloidin staining (Figure 3C,D). The cytoskeleton was rearranged to the cell borders and the F-actin intensity was decreased with the application of ROCK inhibitors. β-catenin was immunostained and clumping of β-catenin was shown in cells treated with Y-27632, sovesudil, and PHP-0961 (Figure 3E).

### 2.4. Cell Signaling

**Figure 4:**
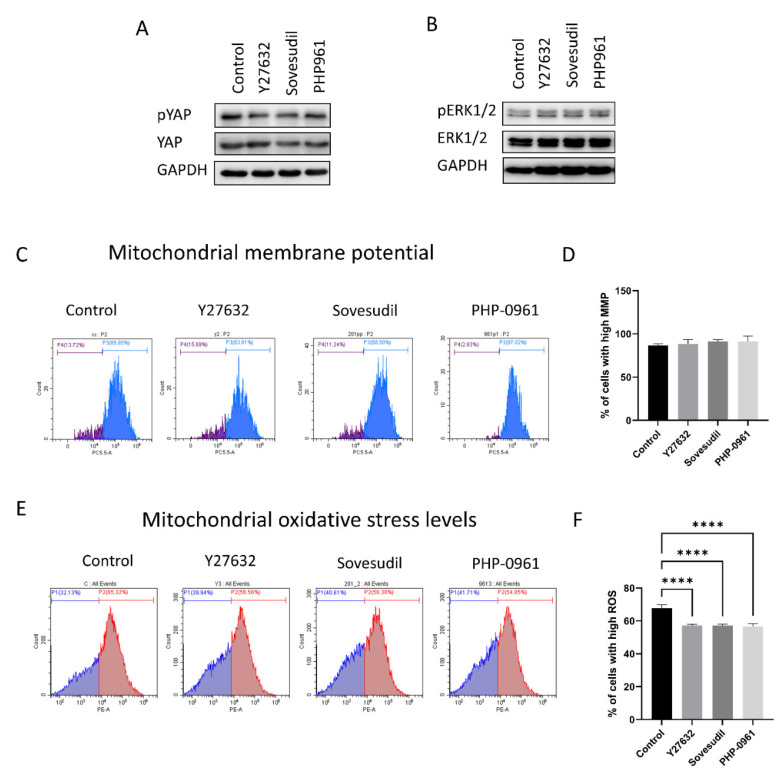
The signaling pathway and mitochondrial function were evaluated. (**A**,**B**) Phosphorylation of YAP and ERK1/2 was evaluated by Western blotting. (**C**,**D**) Mitochondrial membrane potentials were evaluated using the MitoPotential assay kit. (**E**,**F**) Mitochondrial oxidative stress levels were evaluated by MitoSOX red fluorescence intensity. **** *p* < 0.0001.

The signaling pathway including Hippo signaling and ERK1/2 signaling was assessed (Figure 4A,B). All groups with ROCK inhibitors showed that YAP phosphorylation decreased and ERK1/2 was activated compared to the control. Mitochondrial functions were evaluated by mitochondrial membrane potential and mitochondrial oxidative stress levels. Mitochondrial membrane potentials were not changed in Y-27632, sovesudil and PHP-0961 and it was highest in PHP-0961 (Figure 4C,D). Mitochondrial oxidative stress levels were lower in Y-27632, sovesudil, and PHP-0961 (Figure 4E,F).

### 2.5. Porcine Ex Vivo Study

**Figure 5:**
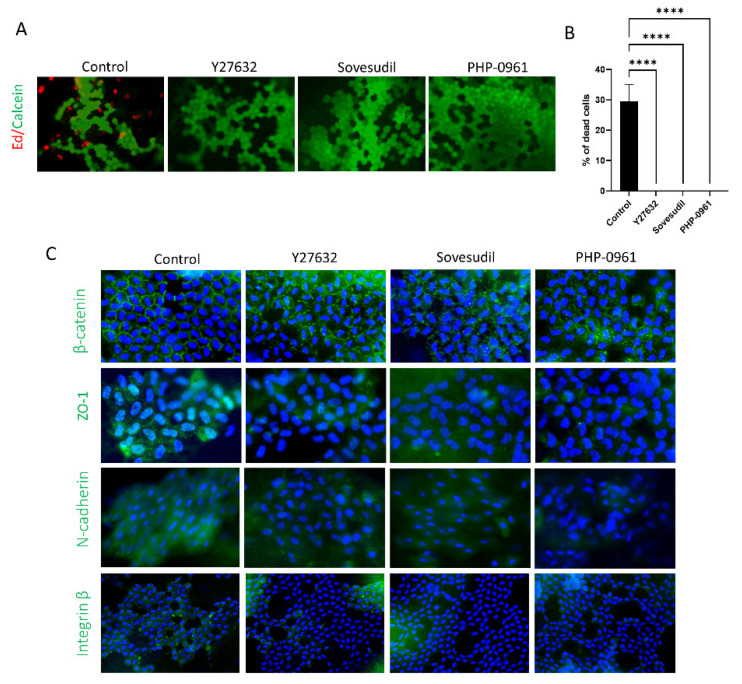
Effect of sovesudil on protection and cell adhesion of corneal endothelium in porcine ex vivo study. (**A**,**B**) Live/dead cell assay. Red is dead, and green indicates live cells. (**C**) Immunofluorescence staining of β-catenin, ZO1, N-cadherin and integrin-β1. **** *p* < 0.0001.

To investigate the effect of Y-27632, sovesudil and PHP-0961 on cell viability of ex vivo porcine cornea, Live/dead cell assay was performed. Live/dead cell assay showed the number of live cells treated with Y-27632, sovesudil, and PHP-0961 were higher compared to control (Figure 5A,B). Porcine corneas were immunostained with against β-catenin, ZO1, N-cadherin and integrin-β1, which indicates cell adhesion molecules (Figure 5C). β-catenin clustering in the cell membrane was observed in corneas treated with Y-27632, sovesudil, and PHP-0961. Expressions of N-cadherin and integrin-β1 were reduced in corneas treated with Y-27632, sovesudil, and PHP-0961.

**Figure 6:**
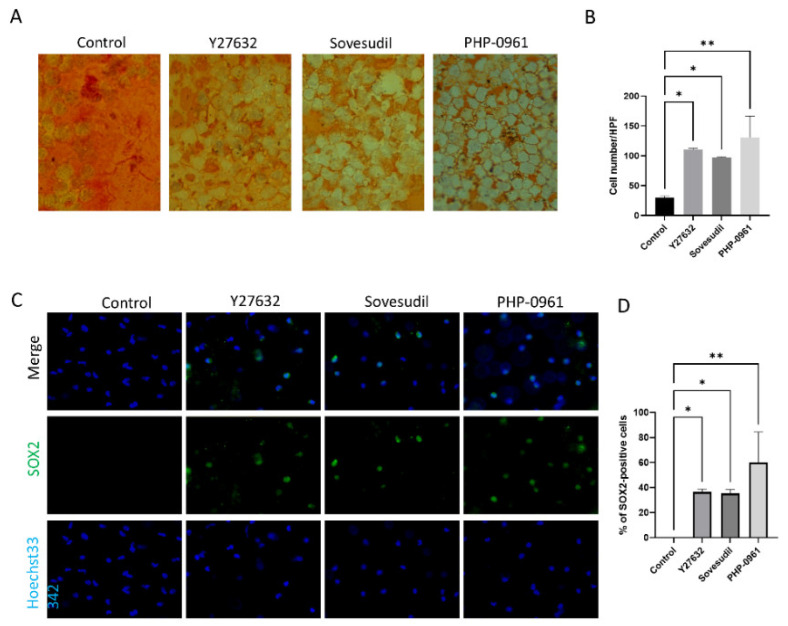
Effect of sovesudil on regeneration of corneal endothelium in a porcine ex vivo study. (**A**,**B**) Alizarin S red staining showed the faster wound healing in corneas with ROCK inhibitors. (**C**,**D**) SOX2 expression was evaluated by immunofluorescence staining of SOX2. * *p* < 0.05 and ** *p* < 0.01.

To evaluate the effect of Y-27632, sovesudil and PHP-0961 on wound healing of corneal endothelium of ex vivo porcine cornea, Alizarin S red staining was performed. Alizarin S red staining showed faster wound healing in porcine corneas with Y-27632, sovesudil, and PHP-0961 (Figure 6A,B). SOX2 was immuno-stained as the proliferation marker. SOX2-positive cells were higher in Y-27632, sovesudil and PHP-0961 (Figure 6C,D).

## 3. Discussion

hCEnCs do not proliferate in vivo, and when there is a large loss of hCEnCs such as in cases of bullous keratopathy, there is no treatment except for corneal transplantation [19]. In this study, we investigated the effect of a novel ROCK inhibitor, sovesudil, on the regeneration of hCEnCs.

Our results show that the novel ROCK inhibitors, sovesudil and PHP-0961, increased cell viability and proliferation, which was confirmed that proliferation marker including SOX2 and Ki67 was increased by sovesudil and PHP-0961. SOX2 is a transcriptional factor that is essential for maintaining self-renewal and proliferation and plays an important role during the development of hCEnCs [5,20,21]. The proliferation marker Ki67 is associated with cell cycles and has been commonly used to evaluate the proliferation [22]. These results are consistent the previous study reporting that ROCK inhibitors promote proliferation of hCEnCs [8,23]. The effect of sovesudil and PHP-0961 could be achieved by providing energy for proliferation through a reduction of mitochondrial oxidative stress levels and an enhancement of mitochondrial function, which is supported by the results shown in this study [24,25,26]. Mitochondrial fission, dysfunction and apoptosis is mediated by ROCK activation through the recruitment of Drp1 into mitochondria [27,28,29]. Novel ROCK inhibitors have the potential to recover mitochondrial function [30]. In this study, phosphorylation of YAP was decreased by the novel ROCK inhibitors. YAP, a member of Hippo signaling pathway, is involved in senescence and regulates proliferation and EMT through TGF-β signaling [31,32]. ROCK drives TEAD/YAP transcription and regulates intracellular tension [33,34]. ROCK inhibitor reduces nuclear YAP/TAZ localization, which regulates senescence [35,36]. Oxidative stress and senescence are reduced via inducing mesenchymal-epithelial transition (MET), and inhibiting TGF-β signaling [16,37]. ERK1/2 regulates the cell proliferation and migration [38].

We evaluated the effect of ROCK inhibitors on cell adhesion using the crystal violet assay. The results showed that cell adhesion was increased by novel ROCK inhibitors. Zonula occludens protein 1 (ZO-1), a peripheral membrane protein expressed in tight junctions, and ZO-1-associated nucleic acid binding proteins (ZONAB) regulate cell proliferation by regulating the nuclear accumulation of CDK4 [39,40]. Both overexpression of ZONAB or knockdown of ZO-1 leads to increased cell proliferation and induces EMT [40,41]. ROCK inhibitor regulates tight junction function and induces the reorganization of apical F-actin [42]. Epithelial cadherin (E-cadherin), a specific component of the epithelial adherens junctions that are integral in cell adhesion, is increased by inhibition of EMT or induction of MET [43,44,45]. ROCK1 inhibitors suppress or reverse EMT by increasing E-cadherin [46]. In this study, N-cadherin and integrin-β1 was not detected. N-cadherin, a member of the calcium-dependent adhesion molecule family of classical cadherins, is upregulated during EMT [43,47]. Integrin-β1 is involved in TGF-β-mediated EMT [48,49]. The results from this study suggests that ROCK inhibitors suppress EMT associated N-cadherin and integrin-β1 expression.

Results from the cell experiments were confirmed with comparable results in the ex vivo porcine cornea study. Experiments using porcine corneas also showed that ROCK inhibitors promote wound healing, cell viability, and adhesion molecules. It is shown by the clustering of β-catenin, and decreased expression of N-cadherin and integrin-β1, results which are consistent with the cell experiments. Previously, it has been reported that inhibition of ROCK signaling induced a morphological change of hCEnC and cell proliferation in human cornea [50]. In addition, the expression of SOX2, which promotes proliferation and stemness [51], was upregulated by the ROCK inhibitors. The expression of the proliferation markers BrdU and Ki67 was also upregulated by the ROCK inhibitors, which is consistent with cell experiments. However, cultured hCEnCs and porcine corneas were used in our study, which are potentially insufficient to reflect the proliferation and healing of hCEnCs in vivo condition. Thus, a future clinical human study is necessary to apply it to clinical treatment.

Different from previous ROCK inhibitors, the novel ROCK inhibitors, sovesudil and PHP-0961, are the soft drugs that is therapeutically active compounds that undergo a predicted fast metabolism into inactive, nontoxic metabolites after exerting their therapeutic effect [12,13,52]. Although it lowers the intraocular pressure and is more stable in anterior chamber [53], there has been no study to report the effect of on regenerate the corneal endothelium. In this study, we compared sovesudil and PHP-0961, the novel ROCK inhibitors, with the positive control Y-27632. Interestingly, the effects of the novel ROCK inhibitors on proliferation and adhesion of hCEnCs were equal to or better than Y-27632. Sovesudil and PHP-0961 are designed to have a higher affinity for ROCK than Y-27632 and sovesudil is soluble in water due to its hydrophilic nature [42]. It was clinically studied for the treatment of glaucoma and these studies established efficacy and safety for use in human patients [42]. Thus, sovesudil may be a novel effective drug that can be used clinically in humans to treat diseases at the front of the eye, such as corneal dystrophies, in addition to glaucoma.

This study shows that YAP, a downstream effector of Hippo signaling, is affected by ROCK inhibitor in the corneal endothelium. YAP in the nucleus controls the cellular responses through interaction with TEAD transcription factors [54]. pYAP proteins are mainly in the cytoplasm and inhibits cellular proliferation by spatially segregating YAP from the nucleus [55]. YAP, a mechanosensor, may be influenced by mechanical force caused by changes in actin filaments [56]. Hippo signaling regulates cell mechanics by controlling focal adhesion assembly [57]. Thus, ROCK inhibitor may cause the change of N-cadherin, E-cadherin and integrin-β1 and the clumping of β-catenin through altering Hippo signaling. β-catenin interacts with α-catenin, which connects the actin cytoskeleton to the adhesion complex [58]. YAP also is regulated by SOX2 to involve in maintaining stemness [59]. This study found that β-catenin at the cell membrane was clumped and the SOX2 levels in the nucleus were elevated by ROCK inhibitors. β-catenin controls E-cadherin-mediated cell adhesion at the plasma membrane and mediates the interplay of adherens junction molecules, which plays an important role in maintaining barrier function [60]. SOX2 inhibits Wnt-β-catenin signaling, inhibits apoptosis and enhances the stemness [61]. The increased expression of SOX2 in this study suggests that the silent corneal endothelial stem cells can be activated by ROCK inhibitor. Furthermore, this study used the organ culture to evaluate and to compare the effect of ROCK inhibitors although there have been several studies using cornea organ culture.

In FECD, hCEnCs are degenerated, mainly due to the formation of guttata in the center of the cornea [62]. The formation of guttata causes corneal edema and subsequently a loss of visual acuity [62]. A potential treatment option to regenerate the corneal endothelium and reduce corneal edema is by removing abnormal central hCEnCs with transcorneal freezing and then applying a ROCK inhibitor, such as sovesudil, to induce the regeneration of peripheral hCEnCs [63,64].

## 4. Materials and Methods

### 4.1. Formatting Cell Culture

This study was performed in accordance with the tenets of the Declaration of Helsinki and was reviewed and approved by the Institutional Review Board/Ethics Committee of Hallym University Kangnam Sacred Heart Hospital (IRB No. NON2020-003). hCEnCs were cultured as previously described [6] and Figure S1. The cornea was purchased from Eversight (Ann Arbor, MI, USA) and the ages of donors ranged from 25 to 32 years. Cells were treated with Y-27632, sovesudil (also known as PHP-201; pH Pharma, Seoul, Republic of Korea), or PHP-0961 (pH Pharma) for 24 h. 

### 4.2. Cell Viability and Toxicity Assay

Cell viability was evaluated using a cell counting kit-8 (CCK-8; Dojindo, Kumamoto, Japan). Briefly, cells (1 × 10^4^) per well are cultured in a 96-well plate and treated with ROCK inhibitors for 24 h. Plates were incubated with CCK-8 solution for 1–2 h. Absorbance was measured at 450 nm, using a microplate spectrophotometer (SynergyHTX, BioTek, Winooski, VT, USA) and expressed as a percentage of the control (100%).

Cell cytotoxicity was evaluated using a LDH assay (Takara Bio; Kusatsu, Japan). Briefly, cells (1 × 10^4^) per well are cultured in a 96-well plate and treated with ROCK inhibitors for 24 h. 50 μL of media were transferred into a new 96 well plate and 50 μL of reaction mixture were added into each well. The plates were then incubated for 30 min. The absorbance at 490 nm was measured using a plate reading spectrophotometer (SynergyHTX).

### 4.3. Proliferation Assessment

BrdU proliferation assay kit (Roche Diagnostics, GmbH, Mannheim, Germany) was used for cell proliferation assessment. Briefly, cells (5 × 10^3^ cells/well) were placed in 96-well plates and incubated for 24 h in a humidified atmosphere containing 5% CO_2_. Cells are treated with ROCK inhibitor and BrdU solution is added to each well and then incubated for 24 h. After removing the media, the plate was incubated in the FixDenat solution for 30 min at room temperature. The plated was then incubated with an anti-BrdU-POD solution for approximately 90 min at 25 °C. Substrate solution is added to each well, and the plate was incubated for 20 min at room temperature. Stop solution was added to each well and the optical density was measured at 450 nm using a microplate reader (Synergy HTX). Proliferation rates were expressed as the percentage of controls after subtraction of the corresponding blanks.

### 4.4. Immunofluorescence Staining

The hCECs were cultured in a slide chamber and treated with ROCK inhibitors and incubated for 24 h. After removing the media, they were washed with phosphate-buffered saline (PBS), and fixed for 20 min in 3.7% (*v*/*v*) formaldehyde solution. Cells were permeabilized for 10 min with 0.5% (*v*/*v*) Triton X-100 and blocked for 1 h with 1% (*w*/*v*) bovine serum albumin (BSA) at room temperature. After washing with PBS, the cells were incubated overnight with either rabbit anti-Ki67 antibody (sc-23900, Santa Cruz, CA, USA), ZO-1 (sc-10804), β-catenin (ab32572), integrin β1 (sc-18887), SOX2 (sc-365823), N-cadherin (sc-59987), or E-cadherin (sc-8426) at 4 °C, and then washed with PBS. Cells are incubated with fluorescein isothiocyanate-conjugated goat anti-rabbit IgG antibody (1:100) or anti-mouse IgG antibody for 1 h at 37 °C in the dark. Cells were counterstained with Hoechst33342 nuclear staining dye. After extensive washing with PBS, the slides were mounted in a drop of mounting medium to reduce photobleaching. Negative control staining was conducted in parallel with the omission of the primary antibodies.

### 4.5. Wound Healing Assay

Cells are cultured at confluence in 12-well culture plates. A wound was scratched with scratcher (SPL) and observed for the migration area. Cells in suspension were washed off with PBS. The culture medium was replaced with medium containing ROCK inhibitors. The distance between the wound, that is, the distance between cells that exist at one edge of the linear defect and those that exist at the opposite edge of the defect after 0 and 24 hrs of incubation.

### 4.6. Cell Adhesion Assay

Cells were allowed to adhere to the culture plate for over 2 or 6 h. The cultured confluent cells were washed twice with PBS and then pretreated 30 min with ROCK inhibitors. Cells were trypsinized, resuspended in their corresponding culture medium, filtered and adjusted to an equal cell number of 2 × 10^5^/mL. Fifty μL of cell suspension was added per well into a 96-well plate. Cells were allowed to adhere to the matrix for 2 or 6 h; nonadherent cells were then washed off with PBS. Cells were fixed with 4% paraformaldehyde for 10 min at RT and stained with 0.1% crystal violet solution for 60 min. Plates were extensively washed with water to remove excessive staining, and the dye was solubilized with 10% acetic acid. Absorbance at 570 nm was quantified on a microplate reader.

Adhesion morphology is assessed on hCEnC cultures by actin staining marker phalloidin. Cells treated with ROCK inhibitors were allowed to adhere to the FNC coating for 6 h and then non-adherent cells were washed off with PBS. Actin was directly stained with Alexa Fluor 488H phalloidin and DAPI nuclear staining and cells were observed under fluorescence microscope (DMi8; Leica, Wetzler, Hesse, Germany).

### 4.7. Western Blot

Radioimmunoprecipitation assay buffer (Biosesang, Seoul, Republic of Korea), containing protease (Sigma-Aldrich, St. Louis, MO, USA) and phosphatase (PhosSTOP; Roche, Basel, Switzerland) inhibitor cocktails, was used to isolate total cellular proteins. Protein concentrations are measured by BCA assay. Western blotting was conducted using standard protocols. 5% skim milk was used for blocking the nonspecific binding for 1 h. Primary antibodies were mouse anti- extracellular signal-regulated protein kinases 1 and 2 (ERK1/2) antibody (sc514302, Santa Cruz, CA, USA, 1:500 dilution), mouse anti-pERK1/2 antibody (sc136521, Santa Cruz, CA, USA, 1:500 dilution), mouse anti-YAP antibody (sc-376830, Santa Cruz, CA, USA, 1:500 dilution), mouse anti-pYAP antibody (PA5-17481, Invitrogen, 1:500 dilution) or rabbit anti-GAPDH antibody (LF-PA0212, Abfrontier, 1:5000 dilution). A horseradish peroxidase (HRP) conjugated secondary antibody and a WEST-Queen™ Western Blot Detection Kit (iNtRON biotechnology, Seongnam, Republic of Korea) were used to detect immunoreactive bands. Data were quantified by video image analysis (Luminograph II, Atto, Tokyo). Protein bands were measured by Image J.

### 4.8. Mitochondrial Assay

The mitochondrial membrane potential was measured using Muse™ MitoPotential assay (Merck Millipore, Guyancourt, France). Briefly, cells are cultured and treated with ROCK inhibitors for 24 h, and then trypsinized and resuspended using 95 μL PBS. Cells were incubated with 5 μL MitoPotential Dye for 20 min at 37 °C in the dark. MitoPotential Dye was used to detect changes in the mitochondrial membrane potential and 7-AAD as an indicator of cell death. The fluorescence intensity was measured using flow cytometry (Cytoflex) at an excitation wavelength of 510 nm and an emission wavelength of 590 nm.

The level of mitochondrial oxidative stress was measured using MitoSOX^TM^ Red (Invitrogen) to measure mitochondrial superoxide production. Cells were cultured and treated with ROCK inhibitors, trypsinized, and resuspended with 95 μL PBS. Cells were incubated with 1 μM MitoSOX^TM^ reagent for 10 min at 37 °C in the dark. The fluorescence intensity was measured using flow cytometry (Cytoflex) at an excitation wavelength of 510 nm and an emission wavelength of 590 nm.

### 4.9. Live/Dead Cell Assay of Porcine Ex Vivo Study

Porcine corneas were purchased locally, scratched and treated with ROCK inhibitor. The live/dead cell assay (Invitrogen) was used for the cell viability and toxicity assay in porcine corneas. Corneas were incubated with ROCK inhibitors for 24 h and washed in PBS, placed endothelial side up in a sterile Petri dish, incubated for 45 min at 37 °C with 100 µL of Hoechst 33342 (10 µM), Ethidium homodimer-I (4 µM) and calcein-AM (2 µM), and then gently rinsed in PBS. A radial cut was performed on corneas and the corneas were flat-mounted. Images were obtained using a microscope (DMi8; Leica). The calcein stained cells are calculated, as was the ratio of viable surface area to total analyzed area. The mortality rate was defined as the number of positive Ethidium nuclei out of the total number of cells.

### 4.10. Immunofluorescence Staining of Porcine Corneas

hCEnCs were cultured in slide chamber and treated with ROCK inhibitors and incubated for 24 h. After removing the media, they were washed with phosphate-buffered saline (PBS), and fixed for 20 min in 3.7% (*v*/*v*) formaldehyde solution. Cells were permeabilized for 10 min with 0.5% (*v*/*v*) Triton X-100 and blocked for 1 h with 1% (*w*/*v*) bovine serum albumin (BSA) at room temperature. After washing with PBS, the cells were incubated overnight with mouse anti-BrdU-antibody (sc-32323, Santa Cruz, CA, USA), rabbit anti- Ki67 antibody (sc-23900, Santa Cruz, CA, USA), mouse anti-SOX2 antibody (sc-365823, Santa Cruz, CA, USA), rabbit ZO-1 antibody (sc-10804, Santa Cruz, CA, USA), rabbit anti-β-catenin antibody (ab32572, Abcam), mouse anti-integrin β1 antibody (sc-18887, Santa Cruz, CA, USA), mouse anti N-cadherin antibody (sc-59987, Santa Cruz, CA, USA), and mouse anti E-cadherin antibody (sc-8426, Santa Cruz, CA, USA) at 4 °C, and then washed with PBS. Cells are incubated with fluorescein isothiocyanate (FITC)-conjugated goat anti-rabbit IgG antibody (1:100) or anti-mouse IgG antibody for 1 h at 37 °C in the dark. The cells were counterstained with DAPI nuclear staining dye. After extensive washing with PBS, the slides were mounted in a drop of mounting medium to reduce photobleaching. Negative control staining was conducted in parallel with the omission of the primary antibodies.

### 4.11. Wound Healing Assay of Porcine Corneas

Corneal endothelial side was scratched for making a wound with plastic pipette tip and was observed once a day until total wound closure of the first cornea. A calibrated endothelial wound, using a 10 μL plastic pipette tip, was performed on whole corneas center. Corneas were then stored at 32 °C in a dry incubator. Every day wound healing was observed after corneas incubation with 0.9% sodium chloride under light microscope until closure.

### 4.12. Alizarin S Red Staining of Porcine Corneas

Corneal endothelial side was stained using alizarin S red after totally wound closure of the first cornea. Glutaraldehyde was used for fixation and the corneal endothelium was observed.

### 4.13. Statistics

Data are expressed as the mean ± standard deviation. Statistical analyses were performed using unpaired Student’s *t*-test for two-group comparisons and one-way analysis of variance (ANOVA), followed by Tukey’s multiple comparison test for more than two groups using GraphPad Prism v.9 (GraphPad Software, San Diego, CA, USA). All experiments were repeated at least three times.

## 5. Conclusions

Across all experiments, the results of sovesudil and PHP-0961 were equal or superior to the results of the ROCK inhibitor positive-control, Y-27632. Novel ROCK inhibitors have the capacity to regenerate hCEnCs by enhancing cell proliferation and adhesion between cells.

## Data Availability

All the data utilized in this study are available upon request to the corresponding author.

## Supplementary Materials

The following supporting information can be downloaded at: https://www.mdpi.com/article/10.3390/ijms232314690/s1, Figure S1: Culture of human corneal endothelial cells.
