# Supplementary material for: Novel ROCK Inhibitors, Sovesudil and PHP-0961, Enhance Proliferation, Adhesion and Migration of Corneal Endothelial Cells
Source: Int J Mol Sci. 2022 Nov 24;23(23):14690. doi: 10.3390/ijms232314690 (PMC9740482; doi:10.3390/ijms232314690)

Figure S1. Cultured human corneal endothelial cells (hCEnCs). (A) Cultured hCEnCs show a mosaic shape similar to the in vivo state. Cells were cultured as follows. Briefly, a complex of the CEC–Descemet's membrane was treated for 10 min in 0.25% trypsin and 0.02% ethylenediaminetetraacetic acid (EDTA) solution. After centrifugation at 1500 rpm for 3 min, cells were seeded in a FNC coating mix (Athena Environmental Sciences, Inc., Baltimore, MD, USA)-coated surfaces of six-well plates. When cells grew to be confluent (about 14–21 days), the cells were digested with 0.25% trypsin/0.02% EDTA and passaged at the dilution of 1: 3. (B) Immunofluorescence staining of zonular occludens (ZO-1) in primarily cultured hCEnCs (P0) were performed. ZO-1 (green) and nuclei (Hoechst 33342, blue) staining of hCEnCs is shown.

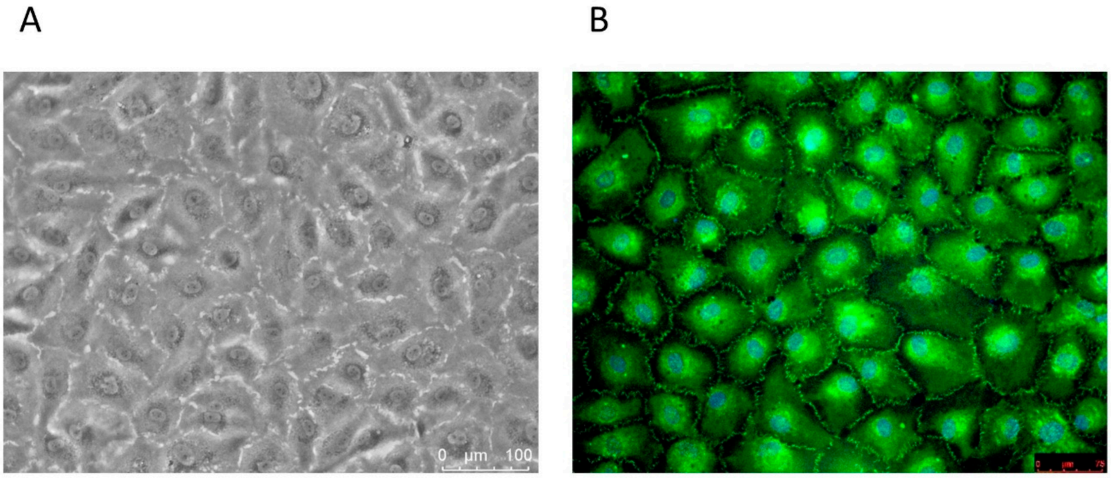

Supplement: Supplementary file 1 [file ijms-23-14690-s001.zip › ijms-1906243-supplementary.pdf]
